# Optimization of Structural Damage Repair with Single and Double-Sided Composite Patches through the Finite Element Analysis and Taguchi Method

**DOI:** 10.3390/ma16041581

**Published:** 2023-02-14

**Authors:** Abdul Aabid, Yasser E. Ibrahim, Meftah Hrairi, Jaffar Syed Mohamed Ali

**Affiliations:** 1 Department of Engineering Management, College of Engineering, Prince Sultan University, P.O. Box 66833, Riyadh 11586, Saudi Arabia; 2 Department of Mechanical and Aerospace Engineering, Faculty of Engineering, International Islamic University Malaysia, P.O. Box 10, Kuala Lumpur 50728, Malaysia

**Keywords:** composite patch, cracked plate, stress intensity factor, finite element method, Taguchi method

## Abstract

Over the last four decades, numerous studies have been conducted on the use of bonded composite repairs for aircraft structures. These studies have explored the repair of damaged plates through experimental, numerical, and analytical methods and have found that bonded composite repairs are effective in controlling crack damage propagation in thin plates. The use of double-sided composite repairs has been found to improve repair performance within certain limits. This study focuses on these limits and optimizes double-sided composite repairs by varying adhesive bond and composite patch parameters. The optimization process begins with a finite element analysis to determine the stress intensity factor (SIF) for various variables and levels, followed by the application of the Taguchi method to find the optimal combination of parameters for maximizing the normalized SIF. In conclusion, we successfully determined the stress intensity factor (SIF) for various variations and normalized it for optimization. An optimization study was then performed using the Taguchi design and the results were analyzed. Our findings demonstrate the repair performance of bonded composite patches using a cost-effective and energy-efficient approach.

## 1. Introduction

The recent success of using externally bonded composite patches to fix fractures and other flaws in aircraft structures has stimulated study in this area. A significant part of the loads acting at the crack tip is carried by the bonded composite repair all through the adhesive, and the presence of the patch thus lowers the stress intensity factor (SIF). Several publications have shown that when the fracture length increases, the mode I SIF of the healed crack exhibits asymptotic behavior [1]. Albedah et al. [2] investigated the effectiveness of single and double circular patches for patching up a fractured airplane structure. The three-dimensional finite element method (FEM) was used to examine the SIF, and it was shown to be in good agreement with the reduction of SIF. Using a combination of evolutionary algorithms and FEM, Ergun et al. [3] investigated the SIF under temperature effect for a patched aluminum plate with a bonded composite patch. Similarly to SIF, the composite patch accurately predicts other fracture parameters, and Kwon et al. [4] employed it to reduce the strain energy release rate (SERR) by repairing the cracked plate on one side. A cracked base plate’s SERR was calculated using an analytical model that was created. Additionally, the stress concentration factors [5,6], J-integral [7,8,9], and fatigue behavior [10,11] have played an important role in fracture parameters, and they have been reduced using a bonded composite patch.

Yala and Megueni [9] were the first to utilize the design of experiments (DOE) method for the optimization of bonded composite repair for the edge-cracked rectangular plate. The DOE approach is used to optimize the impacts of the parameters on the bonded composite repair of an aircraft structure, according to their investigations. However, the parametric research was conducted using SIF, which was computed using the FRANC2D/L finite element code. Later, Fekih et al. [10] modified the composite patch’s parameters to apply a similar idea in order to improve the J-integral performance during repairs. For an edge-cracked plate, the J-integral evaluation was obtained using the ABAQUS finite element code. In order to get a complex combination of parametric effects for an edge-cracked plate, Yala et al. [9] used a full factorial design to obtain a total of 27 simulation outcomes utilizing an L_27_ orthogonal array design. Several studies have been reported to determine the reduction of SIF utilizing FEM and experimental work with an increasing number of composite layers [11] as opposed to analytical work, according to recent publications. The scientists also used several bonded material combinations, such as piezoelectric and composite materials together, to lower SIF [12]. Significant improvements in bonded composite patch repair have been seen in the assessment of bonded composite patches [13]. The Taguchi method is one of the significant approaches to identifying the influenced parameters in any type of linear and non-linear problem, and this can be seen in the experimental work of the glass fibre hybridized flax and sisal fabric hybrid composites to optimize the erosion wear behavior [14].

SIFs were determined for the circular tube with an orientated crack under a uniaxial tensile load. Different results were extracted for the crack tube such as fatigue crack and crack growth curves [15]. A three-dimensional corotational triangle element was developed for density-based topology optimization of thin plate structures with geometric nonlinearity [16]. Imşek et al. [17] performed damage identification in anisotropic-laminated composite beams using sparse modal data and teaching–learning-based optimization. In certain instances, a composite laminate’s mechanical characteristics are anticipated using an approach that combines FEA and machine learning [18]. The FE approach has been used to analyse the adhesive damage for various shapes and types of patches on corroded plates with an angled fracture [19]. Aluminium plates with cracks were subjected to an elastoplastic study with a hybrid repair method that used a bonded composite patch and drilling holes in the opening mode using the FE technique [20]. Additionally, the shape of the composite patch for the repaired and patched Al 2024T3 aircraft plate was done, and its relevance [21] and the behavior of composite patches [22] were identified using the FE approach. 

All the above studies used composite materials to repair the damaged aluminium plate. Whereas composite itself has several advantage and disadvantages and a number of studies has been reported in this area. Some studies investigated the significance of composite materials by considering the different shapes which has been applied the varies loading conditions [23,24]. Also, numerically investigated the energy effect on the impact resistance of an aircraft polymer composite [25]. It is proven that composites has effects of crashworthiness [26] before applying to any object. Therefore, the selection of composite patch materials is important and the current work used the composite material is boron/epoxy which gives more reduction of SIF due to the high shear stress transfer found from the existing work [27,28].

Based on a review of the current literature, only a few studies have been conducted on optimizing composite patch repairs for aircraft structures using the Taguchi method. Most research focuses on a numerical simulation or experimental approach, which can be time-consuming and require extensive analysis to determine the impact of parameters. The Taguchi method offers a useful solution for finding the optimal set of parameters. The goal of this study is to design a finite element model for a center-cracked rectangular aluminium plate under a tensile load and then determine the normalized stress intensity factor (NSIF) using the Taguchi design (L_9_ orthogonal array) for optimization. The parameters considered in this study are the thickness of the composite patch, type of thickness (single or double), adhesive thickness, and cross-sectional area of the patch, which have not been previously defined as a combination.

## 2. Finite Element Method

### 2.1. Geometry and Modelling

**Figure 1:**
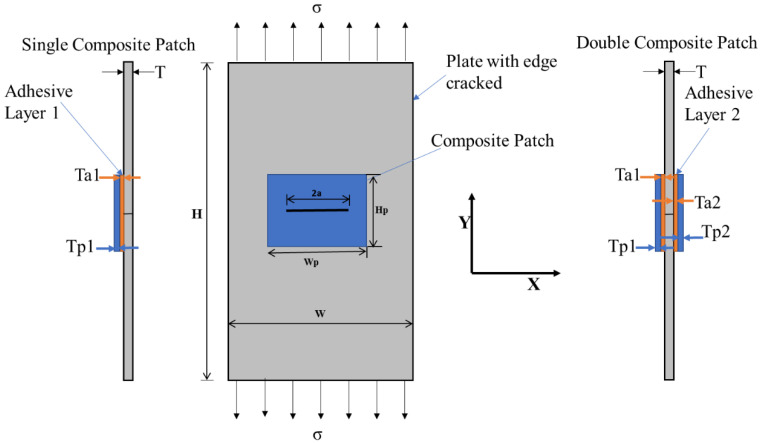
Rectangular plate through a center-cracked integrated composite patch.

Figure 1 displays a rectangular plate with a center crack under tensile stress together with a composite material patch. The composite patch is applied to the crack field to shorten the length of the crack. The length of the crack has been closed by the patch measurements, but the crack area is about twice that. The light weight and high resistance ratio of the composite material are the main criteria. In this instance, boron/epoxy is the composite material that has been used, and it works well to lower the SIF of a rectangular plate that developed a center crack under a tensile load. Because of its outstanding characteristic load transfer, the boron/epoxy patch’s mechanical characteristics were utilized [27,28]. The plate is under plane stress conditions and is exposed to a distant, uniaxial tensile load of 5 MPa. It is only necessary to analyse one-half of the structure because the geometry and loading are symmetric.

The dimension of the plate is a height of 200 mm, a width of 80 mm, and a thickness of 2 mm; the host materials are selected as A2024-T3, which has a high strength-to-weight ratio. Additionally, the patch and adhesive material are chosen as boron/epoxy and FM 73, respectively, and the dimensions of these materials are a height of 20 mm and width of 40 mm, respectively; the thickness of boron/epoxy is 2 mm, and it is assumed that solid material with orthotropic properties and a thickness of the FM 73 adhesive bond is 0.075 mm. The properties of these materials can be seen in Table 1.

### 2.2. Meshing and Boundary Conditions

**Figure 2:**
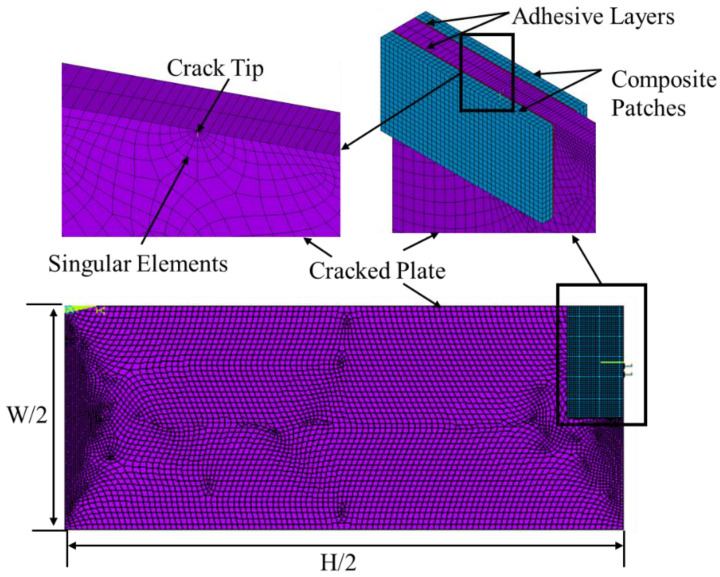
Numerical mesh model.

A program named ANSYS APDL was used to analyse a three-dimensional FE model. Using a three-dimensional SOLID186 element type, a cracked plate, a composite patch, and adhesive bond were all analysed. SOLID186 is one of the 20-node higher-order elements that can be used to analyse solid structures. The singular elements were used at a crack tip to mesh the plate using a free mesh option and produce the SIF. A structured form of mesh with a rectangular shape and an element size of 0.0004 m was used for an adhesive bond and composite patch. Using 18,592, 6249, and 1249 solid elements, respectively, the damaged plate, composite patch, and adhesive bond meshed. With boundary conditions, only a quarter of the model has been modelled for simulation because the model is symmetrical (Figure 2).

### 2.3. Mesh Independence Study

The mesh independence study (MIS) was performed to validate the current simulation model. To validate the current model, existing experimental results were used as benchmark results and compared with it. The three mesh sizes coarse, medium, and fine were considered and compared with each result of the existing experimental work [29]. The size and materials property of the crack damage model was taken from the experimental work of Papadopoulos et al. [29]. Then, one default case was used and was changed into three different mesh sizes based on Table 2. The workstation used in this work is an Intel^®^ Core ™ i9-7980XE CPU operating at 2.60 GHz, 64.0 GB RAM, and a Windows 10-bit operating system, which is available in the Structures and Materials Research Laboratory of Prince Sultan University.

The solution accuracy of the SIF value was improved slightly with the refinement of the mesh from medium to fine, as demonstrated in Table 2. The maximum relative difference was 0.82%. As a result, it was determined to use a medium mesh size for the subsequent simulations due to its adequate resolution and accuracy while reducing the computing time by half. The medium mesh was found to provide sufficient accuracy in the numerical simulation and had a low percentage error of 0.331% compared to the experimental results, as compared to other mesh sizes.

## 3. Taguchi Method

**Figure 3:**
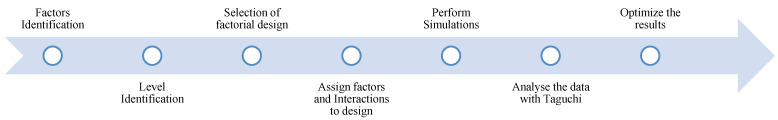
Steps in implementation of the Taguchi approach.

The Taguchi method is used to optimize the current parameters and it is mostly utilized in industries to find the best parameter for improving a performance with the necessary manufacturing process goals [12,13] via the experimental process. Taguchi is the best technique for quickly optimizing the parameters and reducing human efforts on experimentations. Hence, the current work used this concept and performed the FE simulation instead of experiments to predict the present FE results. The parameter considered in this study is the patch’s cross-sectional area (Ap), adhesive thickness (Ta), and composite patch type (TTp). The main goal is to determine the highest NSIF value possible given the parameters. Figure 3 represents the overall Taguchi approach procedure.

Using the Taguchi design of experiments approach, which is a highly effective factorial design method and an orthogonal array (OA) coupled with statistical methodologies, the investigation of parametric optimization of repair of the cracked plate is carried out. An L_9_-OA, which has 9 degrees of freedom and is the most appropriate OA for experimentation/simulations, is taken into consideration in the current work. Table 3 provides illustrations of the selected parameters and Table 4 illustrated a total of nine runs of numerical simulation that were performed based on the selected parameter (P = 3) and their levels (L = 3).

The selected parameters and levels for this study are based on previous research, with a focus on adhesive thickness and the cross-sectional area of the composite patch. It has been established that as adhesive thickness increases, the stress intensity factor (SIF) also increases, and a lower adhesive thickness allows for greater shear stress transfer. As a result, a maximum thickness of 0.09 mm and a minimum of 0.03 mm were chosen. The cross-sectional area of the patch was varied from 225 mm to 400 mm to cover the entire crack area. The minimum level was determined based on the crack length and it is generally recommended that the patch area should be twice the size of the damaged area, leading to the selection of the maximum level. If the patch area exceeds this, debonding may occur. This study introduces a new parameter, patch thickness type, with three different thicknesses being utilized. The first case involves a single-sided patch with a maximum thickness of 2 mm. The other two cases are double-sided composite patches, one of which is based on previous studies and distributes thickness equally between both sides. The other has different thicknesses on each side, offering a new idea to compare and optimize the reduction of SIF.

## 4. Results and Discussion

### 4.1. Determination of Normalized SIF

In the current work, the fracture parameter stress intensity factor is the major consideration of the crack model to analyze the strength of the damaged material. The damaged plate is under a uniform uniaxial tensile stress of 5 MPa, and the dimension considered is very small; therefore, the assumption is made for the present work under plane stress conditions. To optimize the parameters from the selected model, the normalized stress intensity factor was calculated from the ratio of the repaired and unrepaired plate which is subtracted from the value of the tensile load, and is given by

(1) $$K_{N}=\sigma_{p}-\frac{K_{P}}{K}$$

where

$K_{N}$ = Normalized Stress Intensity Factor
$K_{P}$ = Patched Stress Intensity Factor (Repaired)
K = Damaged Plate (Unrepaired)
$\sigma_{p}$ = Applied load.

### 4.2. Finite Element Results

Based on the Taguchi OA/combinations, the results of the NSIF obtained through FEM simulations using ANSYS simulations have been presented in the last column of Table 5. The results indicate a positive impact of bonded composite repair on the reduction of stress intensity factor (SIF). The study evaluated different combinations of bonded composite patch and found variations in the reduction of SIF for each combination. It was observed that the shear stress transformation through the composite patch is effective in reducing crack damage propagation, but the bonded layer must have a thin thickness. The use of double-layer composite patches increases the results, but it also increases the mass gain. To balance the mass gain and improvement in results, the thickness of the double-layer composite patch (DSP 2) was varied differently.

The DSP2 composite patches show a good impact on the reduction of SIF with maintaining the gain weight, whereas DSP1 has good results but the mass gain will also increase. In the case of SSP, whenever the thickness of the composite patch will increase, the SIF will decrease, but the increment in thickness is the weight increment. Based on the present results, it has been found that DSP1 and DSP2 have good results whereas DSP1 needed a thickness of adhesive 0.06 mm while DSP2 can obtain the same results with an adhesive thickness of 0.03 mm. Indeed, it has been proven in previous studies that a lesser amount of thickness will result in a high reduction of SIF [12], and in both cases, the cross-sectional shape of the patch area is the same.

**Figure 4:**
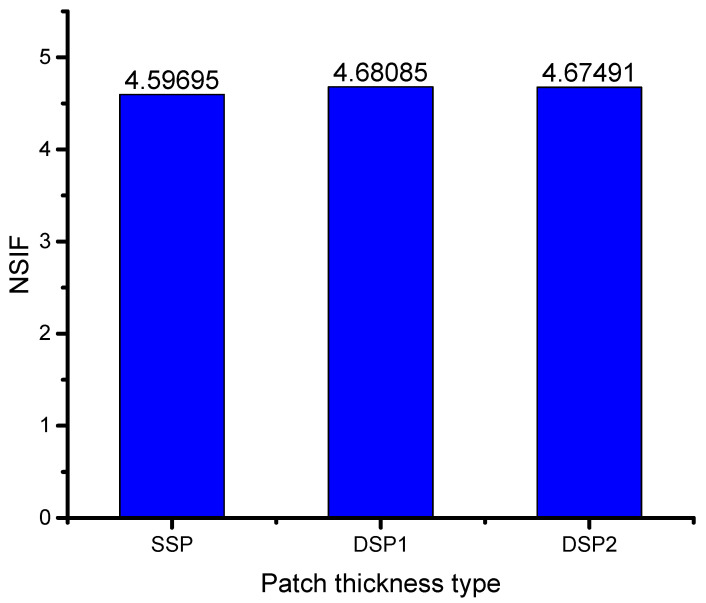
Effect of patch thickness type.

The effect of each type of patch was observed to result in varying the normalized stress intensity factor (NSIF) as shown in Figure 4. The difference between the single and double-sided patch thickness types had a small impact on reducing the NSIF, with the disadvantage of a double-sided patch leading to an increase in component weight. It was also noted that an increase in patch thickness resulted in a decrease in SIF (an increase in NSIF), but the thickness could not exceed the host structure’s thickness to prevent delamination between the patch and plate. In these cases, the adhesive bond study is crucial in determining the bond between the patch and plate. The significance of adhesive thickness was discovered in determining the NSIF. The aluminum and composite materials were bonded using an adhesive, which plays a crucial role in transferring energy from the composite to the cracked aluminum structure. A strong bond between the plate and patch is crucial for the success of the experiment. In the current work, the adhesive bond thickness was modeled using the finite element method and was assumed to be perfectly bonded.

**Figure 5:**
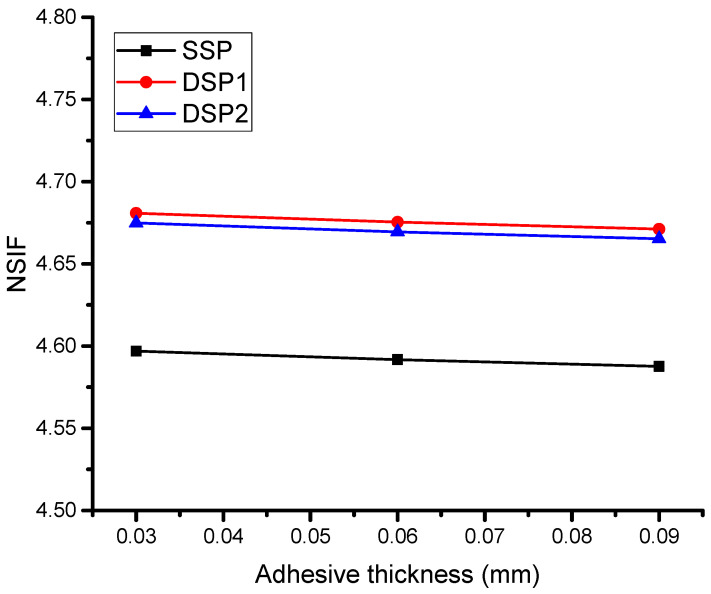
Effect of adhesive thickness.

In order to investigate the bonding effect between the plate and patch, three different thicknesses of adhesive were considered and determined the NSIF, which is shown in Figure 5. Higher NSIF will result in decreasing SIF and this work aims to reduce SIF, which means increasing the NSIF. Figure 5 shows the NSIF has a higher increment when the adhesive thickness has a minimum value. This means the shear stress transfer from the patch to the damaged plate is high and bonding is strong when it is thinner. Indeed, previously, it has been recommended that the adhesive thickness provide a higher reduction in SIF when it is thinner or between 0.25 mm to 0.35 mm [30]. Whenever the thickness of the adhesive bond increases, a small reduction is found in the NSIF. The reduction was not too high, just because the thickness has small changes and the maximum thickness was considered as 0.9 mm, which is smaller than the thickness of the plate as well as patch. However, it is recommended to consider the thickness of the adhesive bond in small amounts for perfect bonding.

The third and crucial parameter of this study is the composite patch size, which has not been adequately addressed in the previous literature. To evaluate the effect of the patch size, three variations have been considered and used for optimization purposes. The ratio of the crack length to the plate width, which varies depending on the orientation of the crack length, determines the strength of the plate. For instance, if the crack length exceeds 50% of the plate width, the crack propagation will be substantial when subjected to a load perpendicular to the crack length. Conversely, if the crack length is less than 50% of the plate width, the control of damage propagation is more effective. The patch dimension also depends on the size of the crack or damaged area, with most studies indicating that the patch size should be twice the crack length. For example, if the crack length is 10 mm, the patch size should be 20 mm, which is also the default value used in this study. However, this study aims to determine the impact of reducing the patch size below double the crack length on reducing crack damage propagation for all three types of patches.

**Figure 6:**
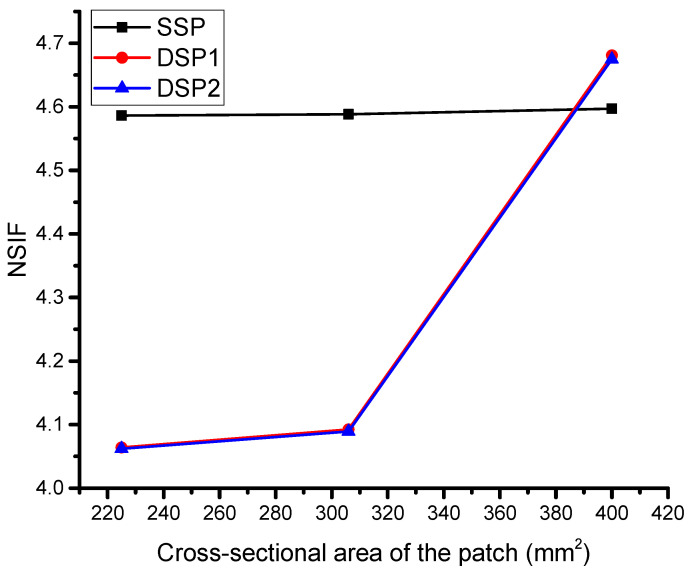
Effect of the cross-sectional area of the patch.

The current study presents a novel aspect of bonded composite repair, which has been overlooked in previous literature. As shown in Figure 6, the SSP (Single-Sided Patch) consistently results in a reduction of SIF (Stress Intensity Factor), with only slight variations in NSIF (Normalized Stress Intensity Factor) when changing the cross-sectional area of the patch. In contrast, DSP1 and DSP2 (Double-Sided Patch) produce results that differ from those of the SSP, indicating that a small patch area will not significantly reduce the SIF unless it is twice the length of the crack.

The study found that a ratio between the crack length and double-sided composite width of 1.5 and 1.75 does not affect the reduction of SIF, as the composite patch on each side transfers shear stress to the crack plate, causing interaction between the bonding and potentially leading to debonding of the plate and patches. The small variations observed in DSP1 and DSP2 can be ignored, as they have similar effects on determining the NSIF. This study also confirms that when the ratio between the crack length and double-sided composite width is 2, there is a substantial reduction in SIF, which is higher compared to the SSP. This is due to the perfect bonding and coverage of the damaged area.

### 4.3. Optimization Results

The most popular method of experimental design to determine the contribution and effects of each chosen parameter is the analysis of variance (ANOVA). The variance analysis for each parameter and a combination of parameters is thus considered first. In this investigation, a 3-level L_9_-OA with nine experimental runs was chosen. The experiment has the total degrees of freedom of 9 + 1 = 8 (Table 6). The interaction effects of each factor and all potential factor combinations are displayed in the ANOVA table. The Minitab 20 and design expert 13 programs were used to create the ANOVA table for an NSIF shown in Table 6. According to Table 6, the cross-sectional area of the composite patch, which has an F-value of 4.38, is the most effective parameter for one-way interactions. This is a result of shear effects on the crack region, and typically, a composite patch area with a higher shear modulus will experience a greater reduction in SIF. The patch type thickness is likely to be effective when it is thicker, and the adhesive’s thickness is lesser, according to the observation of other characteristics.

**Figure 7:**
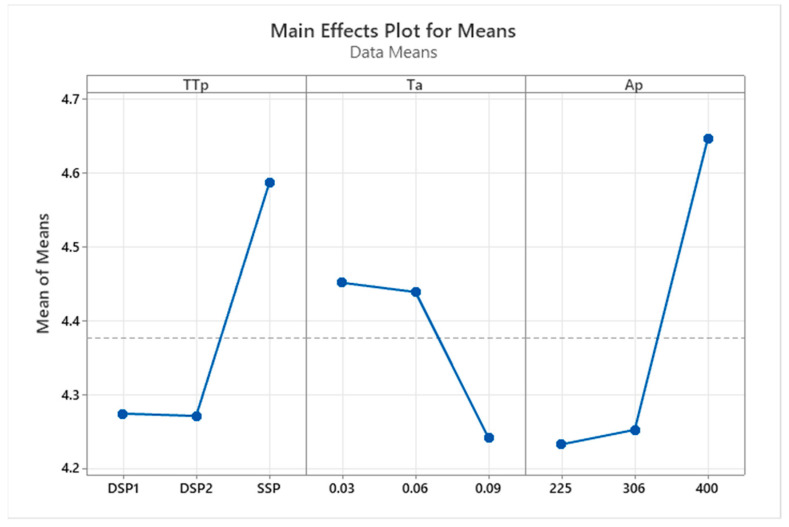
Main effect plot.

A higher F value of 0.33 is discovered in the two-way interactions as a result of the combination of adhesive thickness and the cross-sectional area of the composite patch. This suggests that in the optimization analysis of the current problem, the adhesive thickness and cross-sectional area of the composite patch will be the appropriate combination. In the square term, the cross-sectional area of the composite patch has a good impact on the reduction of SIF with an F value of 1.03 following the adhesive thickness of 0.29. The reasoning behind this is due to all three variables, as well as the utilization of two-square, one-interactions up to the 8-DOF, since there was nothing left to count the error. The outcome of insignificant components and interactions were combined as the effort’s solution. There was a slight variation in the encounter. Its DOF, as well as their SS and MS, have since been grouped. Figure 7 supports these findings, and the main effect plot helps design screening tests. Additionally, the major effects graphs display how each parameter affects the NSIF variance (Figure 7).

A linear polynomial model representing the NSIF as a function of the type of composite patch, the thickness of the adhesive, and the cross-sectional area of the patch can be obtained from the ANOVA analysis and the below regression equation.

NSIF = 4.3771 − 0.1028 TTp_DSP1 − 0.1059 TTp_DSP2 + 0.2087 TTp_SSP + 0.0741 Ta_0.03 + 0.0615 Ta_0.06 − 0.1355 Ta_0.09 − 0.1443 Ap_225 − 0.1246 Ap_306 + 0.2689 Ap_400(2)

**Figure 8:**
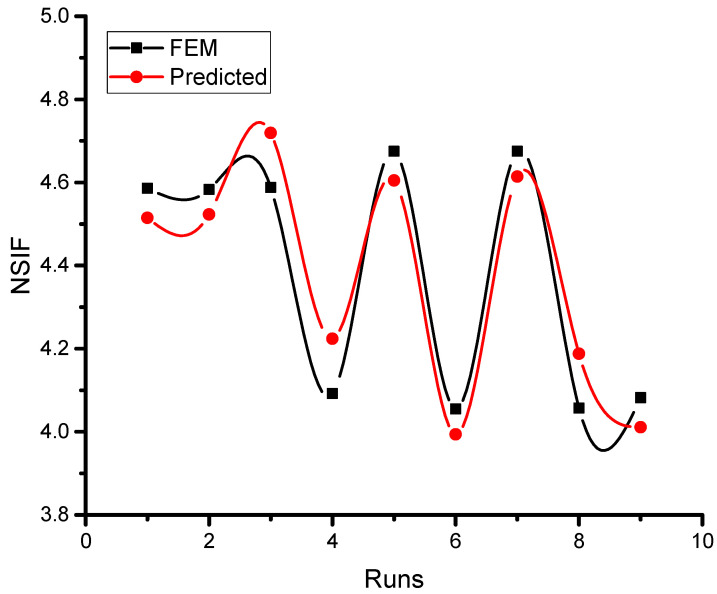
Comparison of FEM and predicted results.

The above Equation (2) has been obtained from the MINITAB tool and Figure 8 displays the NSIF values from the FEM results and the outcomes from the linear polynomial model Equation (2) for nine test instances. According to the FEM and linear expression model results, NSIF will rise as the level of the composite patch area increases. The performance will be marginally impacted by the greater amount of composite patch area paired with other characteristics. For example, it has been discovered that the value of NSIF increases when the simulation case of nine is compared to the case of eight, indicating that the higher level of adhesive thickness will have a lower performance. In fact, it has already been mentioned that a thinner adhesive layer will have a greater impact on NSIF reduction. For all simulation runs, the difference between the FEM results and the outcomes of the anticipated model fit is less than 3.0%. This indicates that the suggested FE model is appropriate for determining NSIF.

**Figure 9:**
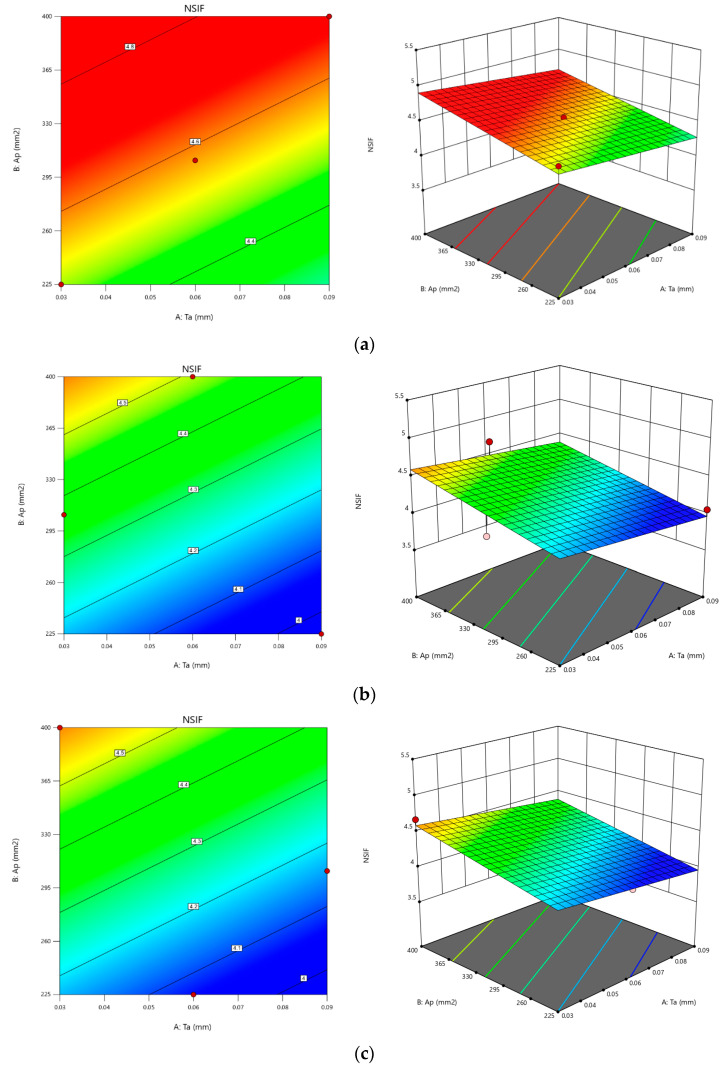
Contours results of NSIF. (**a**) SSP; (**b**) DSP1; (**c**) DSP2.

Figure 9 displays the results of the optimization in the form of contour plots. Figure 9a illustrates that an increase in the composite patch area leads to a higher NSIF (Normalized Stress Intensity Factor) when the patch is bonded on a single side. The impact of the composite patch area and adhesive bond thickness on NSIF is further illustrated in Figure 9b,c for double-sided bonding. The results from each contour are similar; however, the rate of increase in NSIF is higher for single-sided bonding than for double-sided bonding. This suggests that a larger cross-sectional area of the patch and a thinner adhesive bond has a higher rate of shear stress transformation in reducing crack damage propagation for SSP (Single-Sided Patch), while the other end of the damaged plate is less affected as compared to double-sided bonding.

The investigation found that using a double-sided composite patch has a lower significance in reducing crack damage propagation while increasing the patch area and decreasing the adhesive thickness. However, damage control is higher as the damaged plate is bonded on both sides. This can be further increased by increasing the thickness of both sides of the composite, though this also results in increased mass [2]. Moreover, the contour results as per the graphical solution will show the increment and decrement of NSIF concerning the fixed parameters at the x- and y-direction and changes in composite patch type. Thus, the contour results are found to be more informative.

To optimize NSIF, we should choose values for the patch’s cross-sectional area between levels 2 and 3, whereas levels 1 and 3 are the upper and lower limits for adhesive thickness. The highest NSIF values for the composite patch type are found at higher levels. We obtain NSIF values of up to 5.14289 (Table 7) in our optimization prediction table, which is practically impossible given the applied stress of 5 GPa. This optimization, however, needs to be moderated because we also need to take into account other aspects of the patching process. The first of these is the fact that in-plane loading generates out-of-plane bending and highly complex three-dimensional stresses, while excessive shear stress in the adhesive or at the surface of the repaired structure may result in peeling. Consequently, a reasonable and safe value for NSIF should be close to 4.68084 (Table 7). As a result, the composite patch has an adhesive with a thickness of 0.03 mm and a cross-sectional area of 400 mm^2^ with a DSP1 composite patch type. The NSIF derived through FEA simulation for these parametric combinations is 4.88535, which is extremely close to the ideal value discovered during the Taguchi analysis.

## 5. Conclusions

The current study successfully discusses and optimizes the effectiveness of a composite patch on the mode-I stress intensity factor. The finite element method was employed to design and analyze the stress intensity factor of both repaired and unrepaired plates. The model considered a center-cracked rectangular aluminum plate bonded with a composite patch using adhesives. The Taguchi L_9_ orthogonal array was chosen and parameters/levels were set as inputs, including the cross-sectional area of the composite patch, adhesive thickness, and type of composite patch. The response of each parameter was the normalized stress intensity factor. The best-fit parameters were effectively optimized to achieve the optimal result for maximizing the normalized stress intensity factor. The findings suggest that a combination of DSP1 type composite patch, an adhesive thickness of 0.03, and a cross-sectional area of 400 can result in the highest normalized stress intensity factor of 4.68084. The parametric study of the composite patch was concluded to be a significant approach to the mitigating stress intensity factor. 

## Figures and Tables

**Table 1:** Materials properties of the host plate, Boron/Epoxy, and adhesive.

Parameter	Host Plate	Boron/Epoxy	Adhesive FM 73
Poisson’s Ratio (ʋ_12_)	0.33	0.3	
Poisson’s Ratio (ʋ_13_)		0.28	
Poisson’s Ratio (ʋ_23_)		0.28	
Young’s Modulus (E_1_)	68.95 Gpa	210 GPa	
Young’s Modulus (E_2_)		19.6 GPa	
Young’s Modulus (E_3_)		19.6 GPa	
Shear Modulus (G_12_)		7.2 GPa	0.42 GPa (G_a_)
Shear Modulus (G_13_)		5.5 GPa	
Shear Modulus (G_23_)		5.5 GPa	
Density	2715 kg/m^3^	2000 kg/m^3^	1160 kg/m^3^

**Table 2:** Mesh Independence Study.

Type of Mesh	Number of Elements	Number of Nodes	CPU Run Time	Computational Results, SIF KN/m^3/2^	Experimental Result, SIF KN/m^3/2^	Percentage Error
Coarse	13,664	14,786	480 s	58.4	60.2	2.99%
Medium	66,360	69,123	981 s	60.4	60.2	0.331%
Fine	137,840	140,189	1564 s	60.9	60.2	1.149%

**Table 3:** Parameters and Levels (a = 10 mm).

Parameters/Levels	Patch Thickness Type (TTp)	Adhesive Thickness (Ta)	The Cross-Sectional Area of the Patch (Ap)
L1	2/0 (SSP)	0.03	225
L2	1/1 (DSP1)	0.06	306
L3	0.5/1.5 (DSP2)	0.09	400
Units	mm	mm	mm^2^

**Table 4:** Taguchi Orthogonal Array.

Run		1	2	3	4	5	6	7	8	9
Coded Values	A	1	1	1	2	2	2	3	3	3
	B	1	2	3	1	2	3	1	2	3
	C	1	2	3	2	3	1	3	1	2

**Table 5:** Orthogonal array (OA) L_9_ with NSIF results.

Run	Patch Thickness Type (TTp)	Adhesive Thickness (Ta)	The Cross-Sectional Area of the Patch (Ap)	NSIF
1	SSP	0.03	225	4.586136
2	SSP	0.06	306	4.583407
3	SSP	0.09	400	4.587675
4	DSP1	0.03	306	4.092280
5	DSP1	0.06	400	4.675351
6	DSP1	0.09	225	4.055234
7	DSP2	0.03	400	4.674911
8	DSP2	0.06	225	4.056876
9	DSP2	0.09	306	4.081635

**Table 6:** Analysis of Variance (ANOVA).

Source	DF	Adj SS	Adj MS	F-Value	*p*-Value
Model	7	0.62436	0.08919	1.53	0.555
Linear	4	0.48821	0.12205	2.09	0.473
Ta	1	0.06887	0.06887	1.18	0.474
Ap	1	0.25614	0.25614	4.38	0.284
TTp	2	0.16953	0.08476	1.45	0.506
Square	2	0.07578	0.03789	0.65	0.660
Ta*Ta	1	0.01701	0.01701	0.29	0.685
Ap*Ap	1	0.05877	0.05877	1.01	0.499
2-Way Interaction	1	0.01945	0.01945	0.33	0.667
Ta*Ap	1	0.01945	0.01945	0.33	0.667
Error	1	0.05845	0.05845		
Total	8	0.68281			

**Table 7:** Response Optimization: NSIF Parameters.

Variables	Settings	Value		Low Limit	High Limit	
TTp	Free			SSP	DSP2	
Ta	Free			0.03	0.09	
Ap	Free			225	400	
**Response**	**Goal**	**Lower**	**Target**	**Upper**	**Weight**	**Importance**
NSIF	Target	4.05523	4.68084	5.14289	1	1
**Solution**	**TTp**	**Ap**	**Ta**	**NSIF** **Fit**	**Composite** **Desirability**	
1	DSP1	400	0.03	4.68084	1.00000	

## Data Availability

Not applicable.
